# Challenges in economic evaluations in obstetric care: a scoping review and expert opinion

**DOI:** 10.1111/1471-0528.16243

**Published:** 2020-05-05

**Authors:** SM Hulst, WBF Brouwer, BW Mol, ME van den Akker‐van Marle

**Affiliations:** ^1^ Department of Biomedical Data Sciences Section Medical Decision Making Leiden University Medical Centre Leiden The Netherlands; ^2^ Erasmus School of Health Policy & Management Erasmus University Rotterdam Rotterdam The Netherlands; ^3^ Monash Medical Centre Monash University Clayton Vic. Australia

**Keywords:** Economic evaluation, guidelines, obstetric care, scoping review

## Abstract

**Objective:**

The aim of this study is to identify items of economic evaluation guidelines that are frequently not complied within obstetric economic evaluations and to search for reasons for non‐adherence.

**Design:**

Scoping review and qualitative study.

**Setting:**

Literature on economic evaluations in obstetric care and interviews with experts.

**Population or sample:**

The sample included 229 scientific articles and five experts.

**Methods:**

A systematic literature search was performed. All types of literature about economic evaluations in obstetric care were included. The adherence to guidelines was assessed and articles were qualitatively analysed on additional information about reasons for non‐adherence. Issues that arose from the scoping review were discussed with experts.

**Main outcome measures:**

Adherence to guideline items of the included economic evaluations studies. Analytical themes describing reasons for non‐adherence, resulting from qualitative analysis of articles and interviews with experts.

**Results:**

A total of 184 economic evaluations and 45 other type of articles were included. Guideline items frequently not complied with were time horizon, type of economic evaluation and effect measure. Reasons for non‐adherence had to do with paucity of long‐term health data and assessing and combining outcomes for mother and child resulting from obstetric interventions.

**Conclusions:**

This study identified items of guidelines that are frequently not complied with and the reasons behind this. The results are a starting point for a broad consensus building on how to deal with these challenges that can result in special guidance for the conduct of economic evaluations in obstetric care.

**Tweetable abstract:**

Non‐adherence to guidelines in obstetric economic evaluation studies: the difficulties in detail.

## Tweetable abstract

Non‐adherence to guidelines in obstetric economic evaluation studies: the difficulties in detail.

## Introduction

During the last decades, healthcare professionals have been increasingly confronted with rising costs and limited resources. Although new and improved medical interventions can improve health outcomes, resources are scarce, and it is becoming increasingly important for policy makers to make informed decisions on how to spend these resources.

Economic evaluations (EE) provide a framework to compare healthcare interventions in terms of both costs and health outcomes with a relevant comparator, such as standard care. This information can be used by policy makers to decide which interventions maximise total health gains given the scarce resources.^1^

Because these EEs inform such important decisions, many countries have developed guidelines on how to perform them.^2^, ^3^, ^4^, ^5^ These guidelines typically use a reference case to illustrate the main methodological prescriptions which should be adhered to when conducting an EE. The standardisation of methods for EEs is aimed at increase the quality and comparability of the methods across studies, which facilitates decision making.

Guidelines contain recommendations about important characteristics of an EE, such as the viewpoint from which the study is conducted (perspective), the period of time for which cost and effects are assessed, from the beginning of observation to a defined point of time in the future (time horizon) and effect measure. The recommendations apply to healthcare interventions in general. These healthcare interventions, however, are very heterogeneous and, for some, the recommendations may be difficult to comply with.

Previous reviews of EE studies in gynaecology and obstetrics, reported problems with adherence to guidelines,^6^, ^7^ although improvement over time has been observed.^8^, ^9^ However, it was still concluded that improvement of the methodological quality of EEs within the field of obstetrics and gynaecology remained possible and desirable.

Obstetric care has multiple unique features creating methodological challenges in EE. Examples are the combination of the outcomes for mother and child and the assessment of potential long‐term consequences of interventions. These challenges could lead to non‐adherence with standard guidelines. It is currently unclear whether, in obstetrics, researchers deliberately deviate from existing guidelines in order to improve the relevance of their analysis, or they simply fail to comply with the standards for EEs without justification.

The goal of this scoping review of obstetric EEs therefore is to identify items of guidelines frequently not complied with and reasons for such non‐adherence. We focus on the effect side of EEs.

## Methods

### Scoping review

A scoping review was performed^10^ focusing on literature about EEs on interventions directly related to individual obstetric care for women with an ongoing pregnancy, published from the year 2000 onwards. Due to the broad scope of our review, we comprehensively included all types of literature potentially suitable for answering the research question, including EEs, comments, editorials, letters and reviews.

In March 2018, a systematic search was performed in four electronic databases (PubMed, Embase, Web of Science, Cochrane Library) (see Appendix S1). Titles and abstracts were screened according to the criteria listed in Appendix S2. A random sample of 100 articles was screened on titles and abstracts by two authors (SH, MA), resulting in strong agreement. Any disagreements during this process were resolved by discussion. Remaining articles were screened by a single author (SH). In case of doubt, two authors (SH and MA) discussed the article and decided together on inclusion or exclusion.

### Data extraction

Articles were labelled as ‘economic evaluation’, ‘comment/editorial/letter’ or ‘review’. Data extracted from the EEs considered items (see Table 1 for definitions) that are part of reference cases in international guidelines (Appendix S3): the viewpoint from which the study is conducted (perspective), comparator, type of economic evaluation (analytical technique), time horizon, outcome measure used (effects), converting future costs and effects to their present value (discounting) and dealing with unknown information needed for the evaluation (uncertainty). In Appendix S3, guidelines from the Netherlands, UK and USA, the countries from which the majority of the EEs included in this study originate, and a general guideline of Europe^2^, ^3^, ^4^, ^5^ are presented as an example of what most guidelines look like. Most guidelines recommend the same items, although the content of recommendations can differ.^11^

**Table 1:** Definitions of concepts in economic evaluations

Concepts	Definition
Economic evaluation (EE)	The comparative analysis of two or more health interventions in terms of both their costs and consequences. Basic tasks of any economic evaluation are to identify, measure, value and compare costs and consequences of the alternatives being considered
Model‐based economic evaluation (MBEE)	Economic evaluation based on using a model which integrates data from multiple sources
Trial‐based economic evaluation (TBEE)	Economic evaluation conducted alongside a randomised controlled trial (RCT)
Cost‐effectiveness analysis (CEA)	Compares difference in costs of two or more health interventions with the difference in effects. Effects are expressed in a single natural unit, such as ‘neonatal infection prevented’ or ‘preterm birth prevented’
Cost‐utility analysis (CUA)	Compares difference in costs of two or more health interventions with the difference in effects. Effects are expressed in quality‐adjusted life‐years (QALYs)
Quality‐adjusted life‐year (QALY)	QALYs measure health as a function of quality of life and length of life. A life‐year lived in full health is equal to 1 QALY
Utility values	The quality of life is expressed in a utility value, belonging to a certain health state. Utility values of health states range between ‘1’ (full health) and ‘0’ (death) and can be determined by using standardised questionnaires on quality of life, such as the EQ‐5D
EuroQoL‐5 Dimensions (EQ5D)	A standardised questionnaire to measure health‐related quality of life
Perspective	Viewpoint from which the economic evaluation study is conducted (e.g. healthcare perspective or societal perspective)
Comparator	The most used alternative or current practice (usual care)
Time horizon	The period of time for which cost and effects are assessed, from the beginning of observation to a defined point of time in the future
Effect measure	Measure for the effect of the intervention, e.g. natural units (avoided infections) in CEAs or QALYs in CUAs
Discounting	Converting future costs and effects to their present value
Uncertainty analysis	Uncertainty analysis aims at quantifying the sensitivity of the outcome of EE that is due to the uncertainty of the information included in the EE
Reference case	Used by guidelines to illustrate the main methodological prescriptions which should be adhered to when conducting an economic evaluation

The item ‘costs’ was excluded as the methodological choices made on this item were considered less informative for the current study (as being less obstetrics‐specific or a consequence of other choices, such as perspective).

The EEs and other type of articles were also screened for specific information or argumentation about (non‐)adherence to guidelines and reasons behind the methodological choices made. General limitations were not extracted for qualitative analysis, as our aim was to detect specific challenges for EEs in the field of obstetrics.

Qualitative analysis of the retrieved data was done according to the ‘thematic synthesis’ method,^12^ involving three steps. Step 1 comprises free line‐by‐line coding of the findings of primary studies, which means that each line of text is scrutinised for relevant information and subsequently coded according to its meaning and content. Step 2 involves the organisation of these ‘free codes’ into related areas to construct ‘descriptive’ themes. These themes summarise the main subject of the codes, but still stay close to the original content of the data. Step 3 generates analytical themes by identifying underlying themes.^12^ The analytical themes were considered the core outcome of the qualitative analysis.

### Interviews with experts

Subsequently, semi‐structured interviews were held with international experts in the field of EE within obstetric care. The experts could be considered a convenience sample. We approached 10 experts, who were either health economists in the field of obstetrics or obstetricians with experience in health economics. Five of them agreed to participate. These experts elaborated on the analytical themes derived from the qualitative analysis. After briefly introducing each analytical theme, experts were encouraged to elaborate on possible solutions for non‐adherence to guidelines (see interview format in Appendix S4). The interviews were recorded and transcribed. Analysis of the interviews was also performed according to the ‘thematic synthesis’ method.

Due to the methodological nature of this study, no patient public involvement took place. We did not apply for funding for this study.

## Results

### Items of the guidelines most frequently not complied with

The systematic literature search in the scoping review part of this study resulted in 2811 articles considered eligible for screening. We excluded 2474 articles based on title and abstract. The remaining 337 articles were included for full‐text reading. Their references were screened for additional articles (‘snowballing’), which resulted in one additional included article. A total of 229 articles were included for final analysis, including 184 EEs, 25 comments, editorials or letters, and 20 (systematic) reviews (see Figure S1). Of the 184 EEs, 40 were trial‐based (TBEE) (*n* = 36) or cohort studies (*n* = 4), and 144 were model‐based (MBEE). Most studies originated from the USA (*n* = 88), followed by the UK (*n* = 37), the Netherlands (*n* = 23), other European countries (*n* = 15), Australia and New Zealand (*n* = 14), and Canada (*n* = 7). Interventions included screening/diagnostics (*n* = 60), maternal vaccination (*n* = 14), lifestyle intervention (*n* = 12), treatment for maternal disease (*n* = 15), treatment for fetal disease during pregnancy (*n* = 3), treatment for pregnancy‐related pathophysiology (*n* = 27) and interventions related to labour and delivery (*n* = 53).

Table 2 presents the results on methodological choices separately for TBEE and MBEE. We found a wide variety in methodological choices and low adherence to the guidelines for all items except ‘perspective’ and ‘uncertainty’. The low adherence rate for ‘comparator’ was mostly due to a lack of detailed reporting. For example, a lot of articles explained their comparator but did not refer to it as ‘standard care’. We could not link this low adherence to special features of obstetric care. The same goes for ‘discounting’; when the duration of follow up does not exceed 1 year, there is no need for discounting.

**Table 2:** Methodological choices of included economic evaluations (*n* = 184)

Item	Methodological choice	TBEE (*n* = 40)		MBEE (*n* = 144)		Total % adherence^a^ (*n* = 184)	
		*n*	%	*n*	%	*n*	%
**Type of analysis**	Cost‐effectiveness	31	7878	52	36		
	*Cost‐utility*	*3*	*8*	*79*	*55*	*82*	
	Cost‐consequence	2	5	—	—		*53*
	*Cost‐effectiveness and cost‐utility*	*3*	*8*	*13*	*9*	*16*	
	Cost‐effectiveness and cost‐consequence	1	2	—	—		
**Perspective**	*Healthcare*	*25*	*63*	*62*	*43*	*87*	
	*Societal*	*5*	*13*	*51*	*35*	*56*	
	Hospital	4	10	3	2		
	*Third party/healthcare payer*	*1*	*3*	*18*	*13*	*19*	*94*
	*Healthcare and societal*	*5*	*13*	*3*	*2*	*8*	
	*Third party/healthcare payer and societal*	*—*	*—*	*3*	*2*	*3*	
	Unclear	—	—	4	3		
**Comparator**	*Standard care*	*24*	*60*	*92*	*64*	*116*	
	Not referred to standard care, but specified	9	23	38	26		
	Placebo	5	13	1	1		*63*
	Unclear	2	5	13	9		
**Time horizon** ^b^	Short‐term ≤1 year		78	36	25		
	Mid‐term (>1 year, < lifetime)	6	15	9	6		
	*Lifetime maternal*	*—*	*—*	*14*	*10*	*14*	
	*Lifetime neonatal*	*—*	*—*	*48*	*33*	*48*	*49*
	*Lifetime maternal and neonatal*	*—*	*—*	*27*	*19*	*27*	
	*Combination of lifetime and short‐term*	*—*	*—*	*1*	*1*	*1*	
	Unclear	3	8	9	6		
**Effect measure**							
Maternal	Maternal natural units^c^, life‐years or HrQoL^e^	19	48	13	9		
	*Maternal QALYs* ^d^	*3*	*8*	*20*	*14*	*23*	
	*Maternal QALYs* ^d^ *and natural units or life‐years*	*1*	*3*	*2*	*2*	*3*	
Neonatal							
	Neonatal natural units and/or life‐years	7	18	27	19		
	*Neonatal QALYs* ^d^	*—*	*—*	*30*	*21*	*30*	
	*Neonatal QALYs* ^d^ *, natural units and/or life‐years*	*1*	*3*	*8*	*6*	*9*	
Maternal and neonatal							*54*
	Maternal and neonatal natural units, life‐years and/or HrQoL^e^	8	20	12	8		
	*Maternal and neonatal QALYs* ^d^	*—*	*—*	*28*	*19*	*28*	
	*Maternal and neonatal natural units and neonatal QALYs* ^d^	*—*	*—*	*2*	*1*	*2*	
	*Maternal natural units and neonatal QALYs* ^d^	*—*	*—*	*1*	*1*	*1*	
	*Maternal QALYs* ^d^ *, maternal and neonatal natural units*	*1*	*3*	*—*	*—*	*1*	
	*Maternal, neonatal and future offspring QALYs* ^d^	*—*	*—*	*1*	*1*	*1*	
**Discounting**	*Yes, costs and effects equal*	*—*		*67*	*47*	*67*	*58*
	*Yes, costs and effects unequal*	*—*		*11*	*8*	*11*	
	Yes, but only reported costs or effects	3	8	22	16		
	Yes, but unclear	—		1	1		*58*
	*Not, because of short time horizon or other reasons*	*15*	38	*13*	*9*	*28*	
	Not reported	22	55	30	21		
**Uncertainty**	*Deterministic univariate and/or multivariate*	*21*	*53*	*61*	*42*	*82*	
	*Probabilistic (only or combined with deterministic)*	*2*	*5*	*81*	*57*	*83*	
	*Yes, but unclear*	*8*	*20*	*—*	*—*	*8*	*94*
	Not reported	9	23	2	1		

^a^ Methodological choice is in accordance with one or more guidelines (in italics).

^b^ All guidelines mention that the time horizon should be long enough to capture all relevant differences between costs and effects.

^c^ Effects are expressed in a single natural unit, which represents the core medical outcome of a medical trial, such as ‘preterm birth prevented’.

^d^ Quality‐adjusted life‐years.

^e^ Health‐related quality of life.

Only 15% of the TBEEs and 64% of the MBEEs performed a cost‐utility analysis (CUA) as recommended by the guidelines (53% of the total included studies). CUAs are economic evaluations with quality‐adjusted life years (QALYs) as outcome measure. QALYs are determined by multiplying the quality of life (the utility value associated with a health state) by the time spend in that health state. Utility values of health states range between ‘1’ (full health) and ‘0’ (death) and can be determined using standardised questionnaires about quality of life, such as the EuroQoL‐5 Dimensions (EQ‐5D). Therefore, this deviation from the guideline for ‘type of analysis’ also translates into a deviation for effect measures. In TBEEs in particular, the recommended QALYs were rarely used as outcome measures.

Our results show that none of the TBEEs had a lifetime horizon and 63% of the MBEEs adopted a lifetime horizon (53% of the total included studies). Guidelines recommend a lifetime horizon, i.e. cost and effects resulting from the intervention during the whole lifetime should be included, but leave room for other time horizons if well‐argued reasons are provided. In four studies, these reasons were provided, increasing the adherence to guidelines to 5 and 64%, respectively.

### Reasons for non‐adherence: the analytical themes after qualitative analysis

Screening on qualitative information about the methodological choices made, revealed that 70 articles (60 EEs, 5 comments/editorials/letters and 5 reviews) contained information about (non‐)adherence to guidelines, hence being eligible for qualitative analysis.

Following the ‘thematic synthesis’ method, the qualitative data from the EE studies led to two analytical themes, which explain non‐adherence to guideline recommendations on time horizon and type of analysis/effect measure (Table 3):

- Difficulties using the correct time horizon, mainly due to paucity of data on long‐term health effects of perinatal interventions.
- Difficulties in performing a CUA, mainly due to paucity of health‐related quality of life data for obstetric health states and uncertainties in combining utility values for mother and child.

**Table 3:** Analytical themes after qualitative analysis of included articles

	Title of theme	Example of coded information, extracted from included articles
1.	Difficulties deciding on the correct time horizon, mainly due to paucity of data on long‐term health effects of perinatal interventions	*This study did not include longer‐term outcomes for the offspring of pregnant women diagnosed with GDM [gestational diabetes mellitus] because of the paucity of evidence that would link GDM and treatment of GDM to changes in longer‐term outcomes such as obesity and metabolic syndrome in the offspring* ^26^
2.	Difficulties in performing a cost‐utility analysis, mainly due to paucity of health‐related quality of life data for obstetric health states and uncertainties in combining utility values for mother and child	*We are limited by the available utility values for HCV‐related health states. The values that were used in this study were derived from the published literature and originated from a panel of experts who used standard techniques to derive the values. Expert opinion may not reflect the utility values that would be assigned by a patient or the general population.* ^27^ *One methodologic issue of decision and cost‐effectiveness analysis that we explored deserves mention. It is unclear in models of pregnant women whether and how to include the utilities related to the neonate.* ^28^ *An additional, and indeed unique, feature of perinatal care that has received far less attention from analysts conducting economic evaluations surrounds the decision on when to commence ‘counting’ the life of the infant in the calculus. Economic evaluations of perinatal interventions that directly impact prenatal life have typically not incorporated fetal losses into composite measures of health outcome such as LYs or QALYs gained or DALYs averted. As a consequence, miscarriages and stillbirths have not commonly been associated with either health gains or losses. Although the assumption that life commences at birth can be based upon a particular ethical claim or legal understanding, this is rarely, if ever, explicitly stated in published economic evaluations* ^29^

The other type of articles (reviews and comments/editorials/letters) mentioned more common issues. Because of the unspecific character of these qualitative data, this was not summarised in an analytical theme. Some mentioned that often, not all major and relevant health outcomes were considered^13^ or that studies differed in outcome measure used, even when studying the same interventions.^14^ Overall, articles recognised that EE in obstetric care is complicated because outcomes of mother and child are interlinked and that standardisation in this specific area is lacking.

### Integrating the data: challenges of EEs in obstetric care

Integrating the data from the scoping reviews and the interviews with international experts, resulted in the identification of two major challenges and highlighted possible ways to move forward.

#### Challenge 1: Deciding on the correct time horizon

The experts propose that based on clinical evidence, clinicians and health economists can jointly decide on the appropriate time horizon for the analysis. This does not necessarily entail a lifetime horizon but deviating from this should be clearly justified.

Many authors mentioned the lack of and uncertainty about long‐term data on health effects of obstetric interventions as the main reason for choosing a shorter than lifetime horizon. Experts acknowledged that the long‐term follow‐up data of clinical trials are more valid than extrapolating outcomes over longer time periods. However, long‐term data may not always be available, and obtaining it not always feasible, also due to the fact that funders usually have short ‘time horizons’ for grants and will not finance long‐term follow‐up. Suggestions to deal with this problem include using other sources of data and considering new technological options (e.g. with social media) to facilitate gathering follow‐up data on long‐term health effects.

#### Challenge 2: Performing a cost‐utility analysis

TBEEs and MBEEs that did not perform a CUA, and therefore do not use QALYs as their effect measure, mentioned the lack of data on health‐related quality of life (HrQoL) for obstetric and follow‐up health states as the main reason for non‐adherence to the guidelines. With no reliable short‐term and long‐term HrQoL data available, clinical outcome measures were used to determine cost‐effectiveness.

Researchers of TBEEs attempting to collect their own HrQoL data during clinical trials or cohort studies, using standardised instruments such as the EQ5D, pointed out more challenges using QALYs in obstetric care. They mentioned that obstetric interventions often happen within a restricted period, such as the induction of labour, which causes the effects to be too small to result in QALY differences.

Due to paucity of HrQoL data, MBEEs that performed a CUA needed to use best available estimates from the literature or by consulting experts. Many of these studies considered these alternative methods of obtaining utility values a weakness of their analysis and therefore stressed the need for accurate data on quality of life in mothers and infants experiencing obstetric interventions.

Researchers also struggled with combining health effects for mother and child. They raised such questions as whether both maternal and fetal effects should be measured, whether these effects should be combined in one outcome measure, whether the effects in mother and child should be weighted equally, whether utility values of neonates should be included in the maternal health state and whether utility values of other family members should be considered as well.

In the interviews, experts indicated that QALYs might not be an optimal measure, but nonetheless stressed the importance of QALYs as being at least a good generic outcome measure. Although applying the quality of life concept in obstetric care poses challenges, the experts mentioned that when doing a trial, a quality of life measure should be included. As the commonly used instruments may not be suitable in all circumstances, the experts suggested the use of a core outcome set for obstetrics as well as the development of other instruments. For the involved children, measurement of quality of life should start at birth. This could be achieved by asking different sources to indicate the health level (parents, but also clinicians and nurses) and to value these health states.

Experts mentioned that not every cost‐effectiveness analysis (CEA) of an obstetric intervention has to include outcomes in both mother and child, as long as researchers base their decision on clinical evidence, and clearly justify and report their choices. However, when both mother and child are considered, the experts favoured reporting QALYs for mother and child both separately and combined. They argued that if an intervention results in QALY gains for the child but QALY losses for the mother, only reporting the combined outcomes would not be appropriate. Also including other family members might make sense, depending on the situation, according to the experts.

## Discussion

### Main findings

Analysis of 184 EEs of obstetric interventions suggested main areas of current challenges in this field and identified reasons for non‐adherence to guidelines. Researchers seemed to encounter most difficulties with using the correct time horizon and performing a CUA. Most important reasons for non‐adherence appeared to relate to paucity of long‐term health data, difficulties in obtaining data on utility values of health states and combining health effects of mother and child.

### Strengths and limitations

To our knowledge, this was the first study that has qualitatively assessed reasons for non‐adherence to EE guidelines in obstetric care. Although unique in its field, using a scoping review design and qualitative research methods, some limitations need mentioning.

First, because of the scoping character, the amount of included studies can reach considerable numbers.^10^ To restrict the number of articles, articles (*n* = 63) about prenatal screening for fetal abnormalities were excluded, especially as other reviews^15^, ^16^ already discussed the methodological challenges related to EE of prenatal screening interventions. Furthermore, we focussed on CEA/CUA, although cost‐benefit and cost‐minimisation analyses are also forms of health EEs. However, CEA/CUA make up the major part of EEs, and guidelines also focus on these types of analyses.

Second, a scoping study can include all types of literature and study designs, which makes exact replication of this type of review more challenging than in a systematic review conducted according to very strict guidelines.^17^ To reduce potential bias in the review process, inclusion of studies was done according to a pre‐specified and detailed protocol. To confirm correct adherence to the inclusion protocol, a random sample of articles was double‐checked by a second reviewer during the inclusion process.

Third, several methods can be used for qualitative analysis. This study used the ‘thematic synthesis’ method. This method is considered easily applicable, but the flexibility of this method might cause different researchers to choose different aspects of the data as their focus. During our qualitative analysis, two main analytical themes arose. These themes were further explored in the semi‐structured interviews. This may have caused the experts to focus especially on these pre‐set themes. To avoid missing other challenges, all experts were explicitly invited to think about and discuss other challenges of EEs in obstetric care.

In this study, we focused on the specific methodological challenges related to performing EEs in the field of obstetrics. Of course, the more general methodological challenges related to EEs (e.g. large sample size needed to find a significant QALY difference) add to the complexity. Moreover, we did not focus on cost, modelling methods and equity considerations, as these were considered less obstetrics‐specific, which was confirmed by our text search for information and argumentation on non‐adherence to guidelines.

Non‐adherence to guidelines found in our study can also partly be caused by individual studies originating from another jurisdiction or published before the guidelines chosen in this study were published. However, the majority of the EEs included are from countries of which we included the guideline. Furthermore, restricting our analysis to the 80 publications since 2013, revealed comparable or even lower adherence rates (e.g. 55% for type of analysis and 35% for time horizon).

### Interpretation

Whereas previous studies^8^, ^9^ have focused on assessing the methodological quality of individual EEs in obstetric care using a checklist (e.g. CHEERS Statement),^18^ no in‐depth analysis was performed of what makes adherence to guidelines difficult. This makes comparisons with our results difficult.

Our study showed that included EEs varied widely in terms of chosen time horizon. Although 49% of the included studies used a lifetime horizon, the rest did not. An earlier review focusing on the methodological quality of EEs in obstetrics and gynaecology between 1997 and 2009^8^ also highlighted the great variety in applied time horizon, with 29% of the studies using a lifetime horizon.

The most important reason to choose a different time horizon was the lack of and uncertainty regarding long‐term data on health effects of obstetric interventions. Teune et al.^19^ evaluated the follow up after large obstetric clinical trials. They found that only 16% included follow up of the children after discharge from the hospital. This aligns with our findings, indicating that researchers may lack the clinical data to comply with recommendations on applying a lifetime horizon.

Next to the major issue of paucity of HrQoL data, we also found that researchers question the sensitivity and applicability of the QALY measure for obstetric health states. Petrou and Henderson^20^ also point out that current utility measures, such as the EQ5D, lack sensitivity for subtle changes in health caused by perinatal interventions. This is why Gärtner et al.^21^ started developing a birth‐specific utility questionnaire. Besides the challenges in adhering to common guidelines regarding time horizon and in obtaining required health‐related quality of life data, one of the unique features of obstetric care is that outcomes in both mothers and children are relevant. This causes additional complications in performing EEs. D'Souza et al.,^22^ for instance, mentioned this ‘mother–fetus dyad’ as a complicating factor when applying decision analytic modelling. They suggested specific recommendations for clinical decision analysis studies in perinatology, such as recommendations on time horizon and including health outcomes of both mother and fetus.

As our study showed, EEs in obstetric care come with several challenges. Current guidelines do not provide researchers in this area with specific recommendations or advice on how to deal with these challenges. This may have increased the heterogeneity in methodological choices in EEs in obstetrics, which limits their comparability and jeopardises their methodological quality.

A next step therefore is a broad consultation about how to deal with these challenges. Although not all issues are easily solved, this at least will stimulate standardisation of methods, which increases comparability of studies. This is comparable to the efforts that have been made to standardise EEs in the youth sector.^23^ The results may lead to a set of special recommendations for conducting EEs of obstetric interventions. These recommendations should complement national and international guidelines, comparable to the set of recommendations developed in the field of osteoporosis.^24^

Solving underlying problems that cause non‐adherence should remain a priority. Although financially challenging and time‐consuming, one could think of facilitating clinical trials to prolong their follow up or establishing a catalogue of health utility values with data for maternal and neonatal health states after obstetric interventions, comparable to the overview of childhood health utilities.^25^

## Conclusion

This study identified ‘type of analysis’, ‘effect measure’ and ‘time horizon’ as items of EE guidelines that frequently are not complied with in the context of obstetric interventions. Reasons for non‐adherence especially had to do with the paucity of long‐term health and quality of life data in relation to obstetric interventions, as well as the specific challenges in measuring and combining quality of life in health states that involve mother and child. The results of this study could serve a starting point for a broad consensus building on how to deal with these challenges in future EEs in obstetric care.

### Disclosure of interests

Dr Hulst and Dr van den Akker‐van Marle have nothing to disclose. Dr Mol received grants from NHMRC, personal fees from ObsEva, personal fees from Merck Merck KGaA, personal fees from Guerbet and personal fees from iGenomix, outside the submitted work. Dr Brouwer received grants from Consortium of pharmaceutical companies, outside the submitted work. Completed disclosure of interests forms are available to view online as supporting information.

### Contribution to authorship

MEA‐M conceived the review. MEA‐M, SMH and WBFB participated in the development of the design of the review. SMH carried out the searches. SMH and MEA‐M reviewed articles. SMH wrote the draft manuscript. MEA‐M, WBFB and BWM contributed to the development and finalisation of the paper.

### Details of ethics approval

Not applicable.

### Funding

None.

## Supporting information


**Figure S1.** Flowchart of inclusion process.

**Appendix S1.** Search strategy.

**Appendix S2.** Inclusion and exclusion criteria.

**Appendix S3.** Inclusion and exclusion criteria.

**Appendix S4.** Interview format.

Supplementary Material

Supplementary Material

Supplementary Material

Supplementary Material
