# Supplementary figures and images for: Challenges in economic evaluations in obstetric care: a scoping review and expert opinion
Source: BJOG. 2020 May 5;127(11):1399–407. doi: 10.1111/1471-0528.16243 (PMC7539957; doi:10.1111/1471-0528.16243)

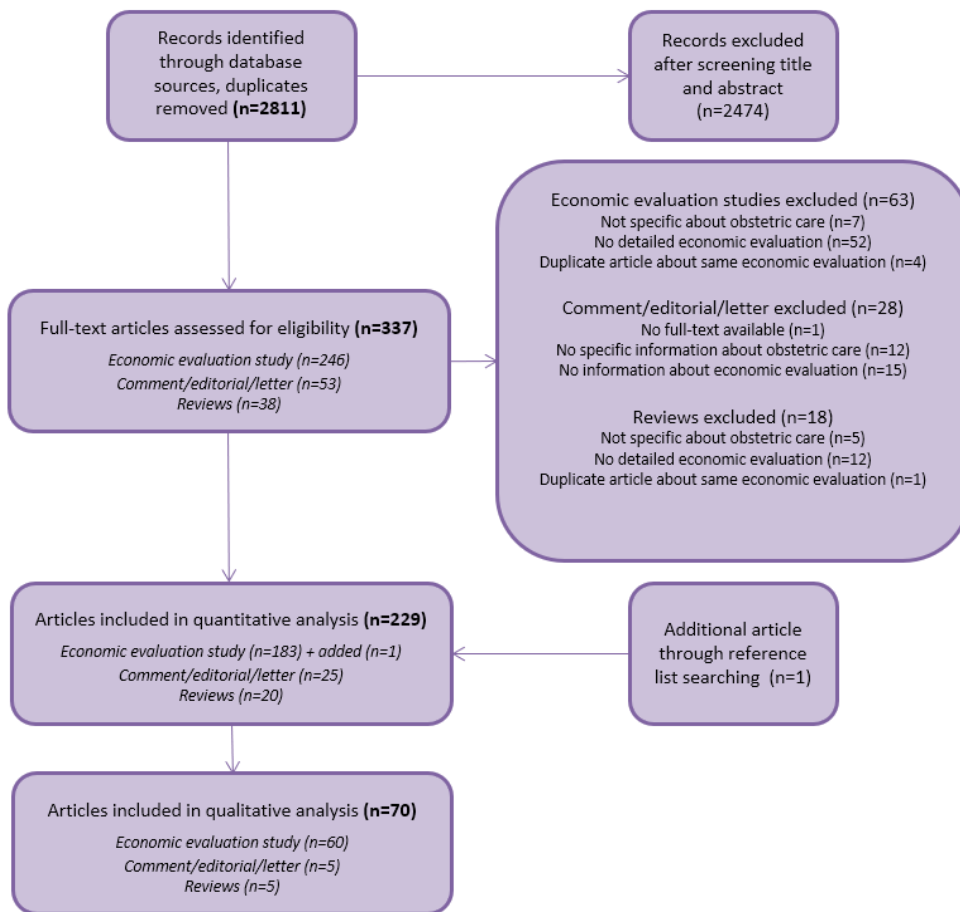

**Figure S1.** Flowchart of inclusion process

Supplement: Supplementary file 1 — Figure S1. Flowchart of inclusion process. [file BJO-127-1399-s001.pdf]
