# Supplementary material for: Challenges in economic evaluations in obstetric care: a scoping review and expert opinion
Source: BJOG. 2020 May 5;127(11):1399–407. doi: 10.1111/1471-0528.16243 (PMC7539957; doi:10.1111/1471-0528.16243)
Supplement: Supplementary file 2 — Appendix S1. Search strategy. [file BJO-127-1399-s002.pdf]

Appendix S1. Search strategy

Search performed on	23-3-2018
Databases	PubMed, Embase, Web of Science, COCHRANE Library
Limit 1-1-2000	2811 (duplicates removed)
Search	**(**(("economic evaluation"[ti] OR "economic evaluations"[ti] OR "economical evaluation"[ti] OR "economical evaluations"[ti] OR "Cost-Benefit Analysis"[majr] OR "Cost-Benefit Analysis"[ti] OR **"Cost Benefit Analyses"[ti] OR "Cost Effectiveness"[ti] OR "Cost Benefit Data"[ti] OR "Cost Utility Analysis"[ti] OR "Cost-Utility Analyses"[ti] OR "Marginal Analysis"[ti] OR "Marginal Analyses"[ti] OR "Cost Benefit"[ti] OR "Costs and Benefits"[ti] OR "Benefits and Costs"[ti] OR "Cost Effectiveness Analysis"[ti] OR Pharmacoeconomic*[ti] OR "economic analysis"[ti]** OR cost effective*[ti] OR ((cost[ti] OR costs[ti]) AND (benefit*[ti] OR utilit*[ti] OR effective*[ti] OR minimisation[ti] OR minimization[ti]))**)** AND ("Pregnancy"[mesh] OR "pregnancy"[tw] OR pregnan*[tw] OR "Gravidity"[tw] OR "Obstetric Labor"[tw] OR "Cervical Ripening"[tw] OR "Labor Onset"[tw] OR "Labor Presentation"[tw] OR "Breech Presentation"[tw] OR "Trial of Labor"[tw] OR "Uterine Contraction"[tw] OR "Maternal-Fetal Exchange"[tw] OR "Parity"[tw] OR "Parturition"[tw] OR "Childbirth"[tw] OR "Childbirths"[tw] OR "Term Birth"[tw] OR "Term Births"[tw] OR "Placentation"[tw] OR "Corpus Luteum Maintenance"[tw] OR "Live Birth"[tw] OR "Stillbirth"[tw] OR "Superfetation"[tw] OR "Pseudopregnancy"[tw] OR "Obstetric Labour"[tw] OR "Labour Onset"[tw] OR "Labour Presentation"[tw] OR "Trial of Labour"[tw] OR "labor stage"[tw] OR "labour stage"[tw] OR "Maternal-Fetal"[tw] OR "Maternal-Foetal"[tw] OR "child birth"[tw] OR "child births"[tw] OR "birth"[tw] OR "births"[tw] OR "Pregnant Women"[mesh] OR "Delivery, Obstetric"[Mesh] OR "Cesarean Section"[tw] OR "cesarean"[tw] OR "C-Section"[tw] OR "Episiotomy"[tw] OR "Obstetrical Extraction"[tw] OR "Obstetrical Vacuum Extraction"[tw] OR "Induced Labor"[tw] OR "induced labour"[tw] OR "Amniotomy"[tw] OR "Fetal Version"[tw] OR "Prenatal Care"[mesh] OR "Prenatal Care"[tw] OR "Obstetrics"[Mesh] OR "Obstetrics and Gynecology Department, Hospital"[Mesh] OR "Obstetric Nursing"[Mesh] OR obstetric*[tw] OR Midwife*[tw] OR Midwive*[tw] OR "Midwifery"[mesh] OR "Maternal Health Services"[mesh] OR "Maternal-Child Health Service"[tw] OR "Maternal-Child Health Services"[tw] OR "Perinatal Care"[tw] OR "Postnatal Care"[tw] OR "Preconception Care"[tw] OR "Pregnancy Complications"[Mesh**]**) NOT ("Animals"[mesh] NOT "Humans"[mesh])) **OR (**(**cost-effective*[tiab] OR** "Cost-Benefit Analysis"[mesh]**)** AND ("Pregnancy"[majr] OR "pregnancy"[ti] OR pregnan*[ti] OR "Gravidity"[ti] OR "Obstetric Labor"[ti] OR "Cervical Ripening"[ti] OR "Labor Onset"[ti] OR "Labor Presentation"[ti] OR "Breech Presentation"[ti] OR "Trial of Labor"[ti] OR "Uterine Contraction"[ti] OR "Maternal-Fetal Exchange"[ti] OR "Parity"[ti] OR "Parturition"[ti] OR "Childbirth"[ti] OR "Childbirths"[ti] OR "Term Birth"[ti] OR

	"Term Births"[ti] OR "Placentation"[ti] OR "Corpus Luteum Maintenance"[ti] OR "Live Birth"[ti] OR "Stillbirth"[ti] OR "Superfetation"[ti] OR "Pseudopregnancy"[ti] OR "Obstetric Labour"[ti] OR "Labour Onset"[ti] OR "Labour Presentation"[ti] OR "Trial of Labour"[ti] OR "labor stage"[ti] OR "labour stage"[ti] OR "Maternal-Fetal"[ti] OR "Maternal-Foetal"[ti] OR "child birth"[ti] OR "child births"[ti] OR "birth"[ti] OR "births"[ti] OR "Pregnant Women"[majr] OR "Delivery, Obstetric"[majr] OR "Cesarean Section"[ti] OR "cesarean"[ti] OR "C-Section"[ti] OR "Episiotomy"[ti] OR "Obstetrical Extraction"[ti] OR "Obstetrical Vacuum Extraction"[ti] OR "Induced Labor"[ti] OR "induced labour"[ti] OR "Amniotomy"[ti] OR "Fetal Version"[ti] OR "Prenatal Care"[majr] OR "Prenatal Care"[ti] OR "Obstetrics"[majr] OR "Obstetrics and Gynecology Department, Hospital"[majr] OR "Obstetric Nursing"[majr] OR obstetric*[ti] OR Midwife*[ti] OR Midwive*[ti] OR "Midwifery"[majr] OR "Maternal Health Services"[majr] OR "Maternal-Child Health Service"[ti] OR "Maternal-Child Health Services"[ti] OR "Perinatal Care"[ti] OR "Postnatal Care"[ti] OR "Preconception Care"[ti] OR "Pregnancy Complications"[majr]) AND ("Randomized Controlled Trial"[ptyp] OR "trial"[tw] OR "RCT"[tw] OR random*[tw] OR systematic[sb]) NOT ("Animals"[mesh] NOT "Humans"[mesh])))
