# Supplementary material for: Challenges in economic evaluations in obstetric care: a scoping review and expert opinion
Source: BJOG. 2020 May 5;127(11):1399–407. doi: 10.1111/1471-0528.16243 (PMC7539957; doi:10.1111/1471-0528.16243)
Supplement: Supplementary file 3 — Appendix S2. Inclusion and exclusion criteria. [file BJO-127-1399-s003.pdf]

Appendix S2. Inclusion and exclusion criteria

Inclusion criteria

1. Published in English and Dutch
2. Published in or after the year 2000
3. The article discussed or performed an economic evaluation in a high resource setting. Studies from USA, Europe, Australia, New Zealand were included.
4. The article discussed or concerned an economic evaluation of an intervention directly related to individual obstetric care for women with an ongoing pregnancy and directly related to obstetric care during labour and delivery (including which health care professional is responsible for the delivery, place of delivery)
5. Articles reporting an economic evaluation study should provide a detailed description of the analysis, comparing cost in monetary terms and effects/consequences in natural units or utilities

Exclusion criteria

1. Studies about reproductive technology/preimplantation genetics
2. Studies about pregnancy complications < 24 weeks. E.g. ectopic pregnancy, miscarriages.
3. Studies about intervention in postnatal period, e.g. breastfeeding and maternity care
4. Studies about contraceptives, abortions, termination of pregnancy
5. Studies about neonatal/paediatric care
6. Studies about national/public health programmes
7. Studies about health policy/implementation of guidelines/management of care
8. Studies about cost saving strategies/economic impact/cost sensitivity/cost-minimization analysis/cost-benefit analysis
9. Studies from Africa/Asia/Latin-America or low/middle income countries, poor/low resource countries
10. Study protocols
