# Supplementary material for: Challenges in economic evaluations in obstetric care: a scoping review and expert opinion
Source: BJOG. 2020 May 5;127(11):1399–407. doi: 10.1111/1471-0528.16243 (PMC7539957; doi:10.1111/1471-0528.16243)
Supplement: Supplementary file 4 — Appendix S3. Inclusion and exclusion criteria. [file BJO-127-1399-s004.pdf]

Appendix S3. Summary of international guidelines

	USA. Guidelines on Cost-effectiveness in Health and medicine 2016 [5]	The Netherlands. Guideline for the Conduct of Economic Evaluations in Health Care 2016 [3]	United Kingdom. Guide to the methods of technology appraisal 2013 [2]	Methods for health economic evaluations - A guideline based on current practices in Europe 2015[4]
**Perspective**	We recommend that all studies report a reference case analysis based on a healthcare sector perspective and another reference case analysis based on a societal perspective.	All relevant societal costs and benefits, irrespective of who bears the costs or to who the benefits go, should be considered in the evaluation and reporting.	NHS and Personal and Social Services	Economic evaluations should at minimum be conducted from a health care perspective. However, several countries require a societal perspective.
**Comparator**	No specific recommendation.	Standard of care or usual care in the Netherlands	As listed in the scope developed by NICE	The guidelines generally seem to agree that at least one of the comparators in an economic analysis should represent those being used in clinical practice.
**Preferred analytic technique**	Cost-effectiveness or cost-utility analysis. *	Cost-utility analysis (CUA)	Cost–utility analysis with fully incremental analysis.	To enhance the usability of the economic evaluations, it is recommended that results be presented in terms of both a cost-effectiveness analysis (CEA) and a cost-utility analysis (CUA).
**Time horizon**	The time horizon adopted in a CEA should be long enough to capture all differences between options in relevant costs and effects.	The time horizon required for an economic evaluation should preferably cover the expected lifetime. A different time horizon may be chosen, however, if well-argued reasons can be provided that a lifetime horizon is not relevant.	Long enough to reflect all important differences in costs or outcomes between the technologies being compared. A time horizon shorter than a patient's lifetime could be justified.	The primary time horizon for the reference case analysis should be sufficiently long to reflect all important relevant differences in costs or outcomes between the technologies being compared.
**Costs**	All resource use should be valued in monetary terms and be included in the numerator of an incremental cost-effectiveness ratio	All costs inside the healthcare sector, patient and family and other sectors. Productivity losses: friction cost method. For reference prices use wherever possible the 'costing guidance' module.	Costs should relate to resources that are under the control of the NHS and personal and social services. These resources should be valued using the prices relevant to the NHS and personal and social services.	To value the resources, the resource use is multiplied with unit costs which depend on the price level in specific countries.

**Effects**	Health effects should be measured in terms of QALYs. In general, we recommend the use of generic preference-based measures such as the EuroQol 5D (EQ-5D), Health Utilities Index (HUI), Short Form 6D (SF-6D), and Quality of Well-Being (QWB).	Expressed in QALYs, a least the EQ-5D-5L with Dutch valuation and whenever relevant also life years gained	Health effects should be expressed in QALYs. The EQ-5D is the preferred measure of health-related quality of life in adults.	The primary outcome measure(s) should where appropriate be presented as natural units (including life-years) and as QALYs.
**Discounting**	Costs and health effects should be discounted at the same rate. 3% is the most appropriate real discount rate for cost effectiveness analyses.	The costs at a constant discount rate of 4%; the future effects at a constant discount rate of 1.5%	The same annual rate for both costs and health effects (currently 3.5%)	Most countries use a discount rate between 3% to 5% for both costs and effects.
**Uncertainty**	Structural uncertainties should be tested in uncertainty analysis. Where there is very little information on a parameter, this should be reflected in a broad range of possible values in the form of a distribution for PSA and a range for deterministic sensitivity analysis.	Uncertainty analyses in empirical economic evaluation: in an empirical approach, subgroup analyses should be performed for all pre-defined subgroups. Uncertainty analyses in a model-based economic evaluation: the influence of parameter uncertainty should be examined using probabilistic sensitivity analyses (PSA) in which all uncertain parameters are analysed.	The impact of structural uncertainty on estimates of cost-effectiveness should be explored by separate analysis of plausible scenarios. Inputs in models must be fully justified and uncertainty explored by sensitivity analysis. Distributions should be assigned to characterise the uncertainty associated with the (precision of) mean parameter values. Probabilistic sensitivity analysis is preferred.	Uncertainty should be explored in sensitivity analyses. To meet the preferences of many of the countries, deterministic as well as probabilistic sensitivity analysis should be conducted.
*The US guideline refers to the type of economic evaluation as 'cost-effectiveness analysis' but states the health effect should be measured in QALYs. According to the four basis types as discussed by Drummond[1], this type of analysis would be referred to as a cost-utility analysis.<br><br>Table updated according to information provided by ISPOR[11]				
