# Supplementary material for: Challenges in economic evaluations in obstetric care: a scoping review and expert opinion
Source: BJOG. 2020 May 5;127(11):1399–407. doi: 10.1111/1471-0528.16243 (PMC7539957; doi:10.1111/1471-0528.16243)
Supplement: Supplementary file 5 — Appendix S4. Interview format. [file BJO-127-1399-s005.pdf]

Appendix S4. Interview format

**General**	
1	How would you describe your own academic profile? This will (anonymously) be noted.
2	What is your own experience with economic evaluation in obstetric care?

**Time horizon**	
Many articles consider their short time horizon as a limitation, because it would underestimate the full effect of the intervention.	
3	Is it necessary to evaluate all interventions over a mother and child's lifetime?
Limited evidence on long term health effects of interventions is often considered as a limitation. If taking long term health effects in to account, is considered important, how can we overcome the problem of limited available data?	
4	Longer follow up of clinical trials, to capture long term effects?
5	Would extrapolation be a good option in obstetrics? And is it feasible? For both mother and child?

**Outcome measures/ utilities**	
The biggest problem in modelling studies performing a cost utility analysis, seems to be the paucity in health related quality of life data for mother and child, regarding obstetric interventions.	
6	How to deal with this paucity of data?
7	How to measure neonatal utility? And when should we start measuring this?
Eleven out of 40 economic evaluation alongside a RCT or cohort, used utilities as an effect measure. These studies mention difficulties with instruments measuring health-related quality of life, because obstetric intervention have effect within a restricted time frame. These effects seem difficult to capture in terms of health related quality of life.	
8	Are QALYs a feasible effect measure in obstetric interventions with a restricted time frame, such as induction of labour ?
Most studies that use utilities, seem to struggle with combining maternal and neonatal utilities. Some argue why they leave maternal or neonatal values out, some weigh them equally in to one outcome measure, some plead for incorporating neonatal utility into the maternal value.	
9	What would be the most appropriate way to combine these outcomes?
10	Do outcomes of other family members have to be considered? If so, which family members?
Because of above mentioned difficulties regarding cost-utility analysis of obstetric interventions, almost half of the included studies chose not to perform a cost-utility analysis.	
11	How to deal with the studies that present their outcomes in natural units?

**Guidelines**	
12	Is there a need for a specific guideline about economic evaluation in obstetric care? And why?
13	What items should first be reached consensus about?
